# Future ozone-related acute excess mortality under climate and population change scenarios in China: A modeling study

**DOI:** 10.1371/journal.pmed.1002598

**Published:** 2018-07-03

**Authors:** Kai Chen, Arlene M. Fiore, Renjie Chen, Leiwen Jiang, Bryan Jones, Alexandra Schneider, Annette Peters, Jun Bi, Haidong Kan, Patrick L. Kinney

**Affiliations:** 1 State Key Laboratory of Pollution Control and Resource Reuse, School of the Environment, Nanjing University, Nanjing, China; 2 Institute of Epidemiology, Helmholtz Zentrum München, Neuherberg, Germany; 3 Department of Earth and Environmental Sciences and Lamont–Doherty Earth Observatory of Columbia University, Palisades, New York, United States of America; 4 Shanghai Key Laboratory of Atmospheric Particle Pollution and Prevention, Shanghai, China; 5 School of Public Health, Key Laboratory of Public Health Safety of the Ministry of Education and Key Laboratory of Health Technology Assessment of the Ministry of Health, Fudan University, Shanghai, China; 6 Asian Demographic Research Institute, School of Sociology and Political Science, Shanghai University, Shanghai, China; 7 National Center for Atmospheric Research, Boulder, Colorado, United States of America; 8 Marxe School of Public and International Affairs, Baruch College, New York, New York, United States of America; 9 Department of Environmental Health, Boston University School of Public Health, Boston, Massachusetts, United States of America; University of Wisconsin, Madison, UNITED STATES

## Abstract

**Background:**

Climate change is likely to further worsen ozone pollution in already heavily polluted areas, leading to increased ozone-related health burdens. However, little evidence exists in China, the world’s largest greenhouse gas emitter and most populated country. As China is embracing an aging population with changing population size and falling age-standardized mortality rates, the potential impact of population change on ozone-related health burdens is unclear. Moreover, little is known about the seasonal variation of ozone-related health burdens under climate change. We aimed to assess near-term (mid-21st century) future annual and seasonal excess mortality from short-term exposure to ambient ozone in 104 Chinese cities under 2 climate and emission change scenarios and 6 population change scenarios.

**Methods and findings:**

We collected historical ambient ozone observations, population change projections, and baseline mortality rates in 104 cities across China during April 27, 2013, to October 31, 2015 (2013–2015), which included approximately 13% of the total population of mainland China. Using historical ozone monitoring data, we performed bias correction and spatially downscaled future ozone projections at a coarse spatial resolution (2.0° × 2.5°) for the period April 27, 2053, to October 31, 2055 (2053–2055), from a global chemistry–climate model to a fine spatial resolution (0.25° × 0.25°) under 2 Intergovernmental Panel on Climate Change Representative Concentration Pathways (RCPs): RCP4.5, a moderate global warming and emission scenario where global warming is between 1.5°C and 2.0°C, and RCP8.5, a high global warming and emission scenario where global warming exceeds 2.0°C. We then estimated the future annual and seasonal ozone-related acute excess mortality attributable to both climate and population changes using cause-specific, age-group-specific, and season-specific concentration–response functions (CRFs). We used Monte Carlo simulations to obtain empirical confidence intervals (eCIs), quantifying the uncertainty in CRFs and the variability across ensemble members (i.e., 3 predictions of future climate and air quality from slightly different starting conditions) of the global model. Estimates of future changes in annual ozone-related mortality are sensitive to the choice of global warming and emission scenario, decreasing under RCP4.5 (−24.0%) due to declining ozone precursor emissions but increasing under RCP8.5 (10.7%) due to warming climate in 2053–2055 relative to 2013–2015. Higher ambient ozone occurs under the high global warming and emission scenario (RCP8.5), leading to an excess 1,476 (95% eCI: 898 to 2,977) non-accidental deaths per year in 2053–2055 relative to 2013–2015. Future ozone-related acute excess mortality from cardiovascular diseases was 5–8 times greater than that from respiratory diseases. Ozone concentrations increase by 15.1 parts per billion (10^−9^) in colder months (November to April), contributing to a net yearly increase of 22.3% (95% eCI: 7.7% to 35.4%) in ozone-related mortality under RCP8.5. An aging population, with the proportion of the population aged 65 years and above increased from 8% in 2010 to 24%–33% in 2050, will substantially amplify future ozone-related mortality, leading to a net increase of 23,838 to 78,560 deaths (110% to 363%). Our analysis was mainly limited by using a single global chemistry–climate model and the statistical downscaling approach to project ozone changes under climate change.

**Conclusions:**

Our analysis shows increased future ozone-related acute excess mortality under the high global warming and emission scenario RCP8.5 for an aging population in China. Comparison with the lower global warming and emission scenario RCP4.5 suggests that climate change mitigation measures are needed to prevent a rising health burden from exposure to ambient ozone pollution in China.

## Abstract 2

Patrick Kinney and colleagues model seasonal and annual future ozone-associated mortality in 104 Chinese cities with different population sizes

## Author summary

**Why was this study done?:**

- Climate change is likely to increase ozone-attributed health burdens, which are not well understood in China, the world’s largest greenhouse gas emitter, with a rapidly aging population.
- Few published studies have jointly evaluated future ozone-related mortality considering changes in climate, population size, population aging, and age-group-specific mortality rates.
- Prior research has mainly investigated summertime ozone, but little is known about the potential seasonal variations of ozone-related acute excess mortality under climate change.

**What did the researchers do and find?:**

- We used a global chemistry–climate model and recently available ambient ozone monitoring data to show future changes in ozone-related acute excess mortality from 2013–2015 to 2053–2055 under different climate and population change scenarios in 104 cities across China.
- We show that global climate change, particularly rising methane, leads to a 10.7% increase in annual ozone-related non-accidental deaths under a high global warming and emission scenario but a 24.0% decrease under a lower global warming and emission scenario.
- Although population size is expected to decline and age-group-specific mortality rates are expected to decrease, population aging offsets these decreases and yields 1–4 times greater ozone-related mortality under climate change in 2053–2055 than that in 2013–2015.
- Ozone concentration increases in the cold season (November to April) under a high global warming and emission scenario, leading to a net increase of ozone-related mortality in 2053–2055.

**What do these findings mean?:**

- To our knowledge, this is the first study to quantify the amplifying effect of population aging on ozone-related mortality under alternative scenarios of climate and population change while accounting for decreases in population size and age-group-specific mortality rates.
- Cold season ozone may play a more important role than currently recognized in future ozone-related mortality burden attributable to climate change, and future studies on seasonal patterns are warranted.
- This study suggests that the ozone-related mortality burden in China will rise under a high global warming and emission scenario of climate change, and mitigation measures are needed to reduce the associated health burden.

## Introduction

Climate change threatens human health via multiple pathways, including both direct impacts from changes in temperature, precipitation, and frequency of extreme weather and indirect impacts mediated through both natural systems (e.g., air quality and disease vectors) and human systems (e.g., undernutrition and mental stress) [1]. Climate change is expected to worsen ozone pollution in already heavily polluted areas through meteorological conditions conductive to the accumulation of air pollution and weather-sensitive natural and anthropogenic emissions of ozone precursors (e.g., more emissions owing to increased air conditioning usage in a warmer climate) [2–6]. Global and regional health impact assessments have found adverse impacts of climate change on ozone-related health burdens [7–17]. However, most of the regional studies have focused on developed countries [7–14], while few studies exist in the largest developing country—China [16,17]. In addition, previous studies applied coarse-scale ozone projections in China [16,17], which may bias ozone-related acute excess mortality in populous urban areas. Punger and West found that using coarse resolutions may lead to a small overestimation (<6%) in the US [18], whereas Fenech et al. found low but significant biases (−0.9% to +2.6%) in the attributable fraction of total mortality due to warm season ozone exposure in Europe [19]. As China is the world’s largest emitter of greenhouse gases [20], and suffers from severe ambient ozone pollution [21], estimating the impact of climate change on ozone-related health effects is critical for understanding the indirect health impacts of climate change in China.

Future ozone-related health burdens will reflect both the impact of changing ozone concentrations and changes in the size and age distribution of the exposed population. Previous studies on ozone-related health impacts under climate change have been limited by only considering changes in population size [9–11,13,14,17], neglecting changes in population aging. This may underestimate the influence of population change since older people are more vulnerable to short-term ozone exposure [22] and have substantially higher baseline mortality rates than younger people [23]. As the age-standardized death rate has been decreasing in China [24], it remains unclear the extent to which population aging may offset the decrease in future ozone-related acute excess mortality due to reduction in the age-standardized death rate.

Little is known about the seasonal variation of ozone-related health burdens under climate change. Most previous studies only projected ozone-related acute excess mortality in the warm season, resulting in an incomplete understanding of the ozone-related acute excess mortality across the year [3,25]. Rising ozone in the non-warm season would also pose risks since epidemiological evidence generally indicates a no-threshold concentration–response relationship between short-term ozone exposure and mortality [26,27]. Moreover, the seasonal cycle of ambient ozone at northern mid-latitudes has already shifted from a summer maximum to a maximum earlier in the year during recent decades [28]. The high-ozone season may continue to shift towards spring and winter under climate and emission changes [29], and the differences in ozone levels between the warm season and the cold season may diminish in the future [30].

In this study, we aimed to estimate near-term future (mid-21st century) changes in annual and seasonal ozone-related acute excess mortality in China attributable to climate and emission change and population change, based on ozone projections from a global chemistry–climate model statistically downscaled to a fine spatial resolution (0.25° × 0.25°) using recently available ambient ozone monitoring data from across China. We estimated the near-term future changes because of their importance to decision makers in government and industry for acting on mitigation and adaptation to reduce the health impacts of climate change. We used 3 ensemble members from the coupled chemistry–climate model in each of 2 climate and emission change scenarios and 6 population scenarios to account for potential uncertainties in climate models, greenhouse gas and pollution emission projections, and population change models [10,23,25].

## Methods

**Fig 1:**
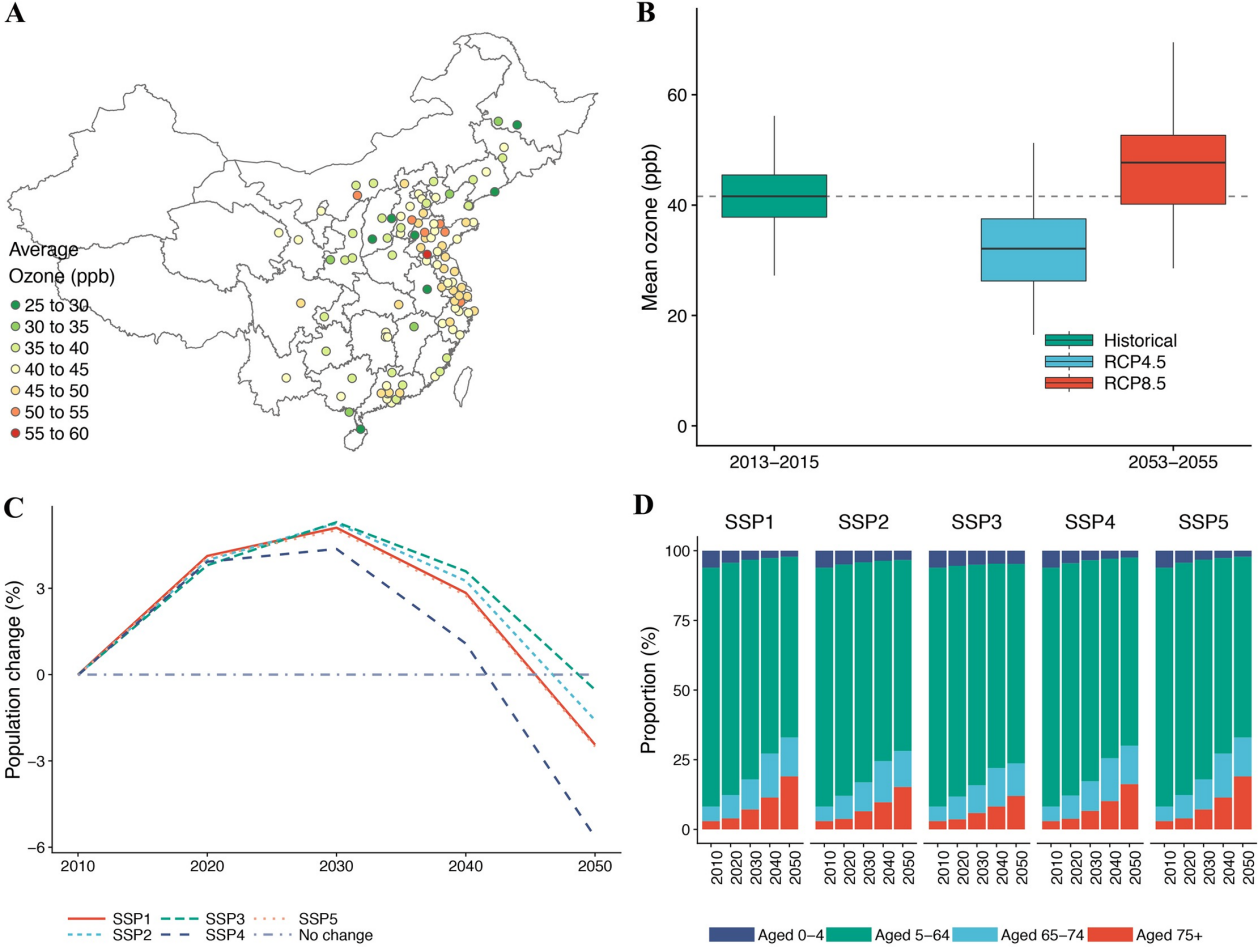
Historical and future changes in ambient ozone concentration and population. (A) Spatial distribution of annual average daily ozone concentration (parts per billion [ppb]) during 2013–2015 (historical period) in 104 Chinese cities. Ozone concentration is the maximum daily 8-hour average. (B) Historical (2013–2015) and projected (2053–2055) annual average daily ozone concentration (ppb) in 104 Chinese cities under the RCP4.5 and RCP8.5 scenarios. RCP4.5 and RCP8.5 represent moderate and high global warming and emission scenarios, respectively. The horizontal line within each box represents the median concentration among 104 cities, the lower and upper boundaries of the box indicate the 25th and 75th percentiles, and the ends of the whisker lines indicate the maximum and minimum concentrations within 1.5 times the interquartile range from the upper and lower box boundaries. (C) Projected population size in 104 Chinese cities from 2010 to 2050 under 6 population change scenarios under different shared socioeconomic pathways (SSPs). (D) Projected population aging in China from 2010 to 2050 under 5 SSP population change scenarios.

This analysis was conducted in 104 Chinese cities (Fig 1A) where historical ozone concentrations, future ozone projections, population change projections, and baseline daily mortality counts were available. These 104 cities are distributed over 29 of the 31 mainland provincial administrative regions and included a total of 170 million people in 2010, accounting for approximately 13% of the total population of mainland China.

### Historical ozone observations

Ambient ozone observations were obtained from the China National Environmental Monitoring Center (http://106.37.208.233:20035). Since ozone was not nationally monitored in China before 2013 and air quality data were publicly available only after 2013, a total of 918 days from April 27, 2013, to October 31, 2015, were chosen to represent the historical period. Correspondingly, the period April 27, 2053, to October 31, 2055, was used to represent the future period. There were 778 national air quality monitoring sites (S1 Fig) involved in this study to compute the historical ozone concentrations and to statistically downscale the future ozone projections. Maximum daily 8-hour average (MDA8) ozone concentrations for each site were calculated from the hourly concentrations for days with at least 75% available values within the day. The MDA8 metric was applied in this study as it reflects more biologically relevant exposure time within a day than the daily 24-hour average metric [31]. During our study period, the 104 Chinese cities had an average of 10.6% of days with missing MDA8 ozone observations. Missing data were not imputed.

### Future ozone projections

We analyzed global ozone simulations performed with the Geophysical Fluid Dynamics Laboratory chemistry–climate model CM3 (GFDL-CM3), which were conducted under the Coupled Model Intercomparison Project Phase 5 (CMIP5), in support of the Intergovernmental Panel on Climate Change Fifth Assessment Report [32]. GFDL-CM3 is a coupled global-scale physical climate model with a comprehensive treatment of interactive tropospheric and stratospheric chemistry, as well as aerosol–cloud interactions and chemistry–climate interactions [33]. GFDL-CM3 has a native c48 cubed sphere horizontal resolution, regridded to 2.0° × 2.5° (latitude × longitude) for our analysis, and includes 48 vertical layers corresponding to the hybrid sigma-pressure levels extending from the surface to about 80 km altitude [33]. A detailed description of the gas-phase chemistry, historical and projected anthropogenic emissions, and lower chemical boundary conditions can be found in Naik et al. [34].

The Representative Concentration Pathway (RCP) scenarios RCP4.5 and RCP8.5 were used to evaluate the future global change, from both well-mixed greenhouse gases and emissions of air pollutants and their precursors, in ozone concentrations, representing moderate and high global warming and emission scenarios, respectively [35,36]. RCP8.5 is a high greenhouse gas emission scenario of without any mitigation [36], whereas RCP4.5 corresponds to a moderate emission scenario [35]. By the end of the 21st century, global warming is likely to exceed 2°C under RCP8.5 and to be between 1.5°C and 2.0°C under RCP4.5 [37]. For each scenario, 3 ensemble members (i.e., 3 predictions of future climate and air quality from slightly different starting conditions) provide some measure of unforced internal variability induced by the chaotic climate system [38]. The analysis of these 2 RCP scenarios enables us to evaluate changes in future ozone air quality in China due to the combined changes in emissions and climate.

Compared with observations, daily GFDL-CM3 simulations overestimated ozone concentrations in the historical period by an average of +17 ppb (36%) across ensemble members. We used a bias-correction spatial disaggregation (BCSD) method to downscale the GFDL-CM3 simulations to a high spatial resolution of 0.25° × 0.25° (approximately 25 km × 25 km). Briefly, in the bias-correction step, we first compared the daily GFDL-CM3 simulations with corresponding daily ozone observations within each coarse-scale grid cell (2.0° × 2.5°) and each month to identify the monthly bias at each quantile in the historical simulations relative to observations. We matched the historical simulations and observations within the same month rather than on the same day to obtain the historical monthly biases. We then corrected model estimates in the future periods, assuming that historical monthly biases persist in the future. We further downscaled the bias-corrected projections to the high spatial resolution (0.25° × 0.25°) using a spatial disaggregation method. In short, we spatially translated the bias-corrected future model simulations from the coarse scale (2.0° × 2.5°) to the fine scale (0.25° × 0.25°). Using the spatial distribution of monthly mean ozone observations as a spatial guide, we first subtracted the coarse-scale spatial distribution of observations from coarse-scale bias-corrected projections to get temporal scaling factors. Then we added the fine-scale spatial distribution of observations back to the fine-scale temporal scaling factors that were interpolated using a bilinear interpolation method. See S1 Method for further details. We computed city-level ozone projections, as well as historical observations, by taking the weighted mean MDA8 ozone concentrations of each grid cell that fell within a certain city boundary.

### Population change projections

To explore the sensitivity of ozone-related acute excess mortality estimates to assumptions about population change, 6 population scenarios were applied in this analysis: a no population change scenario assuming that the Chinese population remains the same from 2010 to 2050, and 5 population projections under the shared socioeconomic pathways (SSPs) in China. For the former, city-level population data were collected from the 2010 Population Census of China. The latter 5 scenarios are drawn from the SSPs, which describe a set of plausible alternative futures of societal development without considering the effects of climate change and new climate policies over the 21st century [39,40]. It is possible to combine SSPs with different RCPs to generate a set of future scenarios that represent different levels of socioeconomic development and climate change [41]. For China, the demographic assumptions of the 5 SSPs are as follows: SSP1 assumes low fertility, low mortality, medium migration, high education, and fast urbanization; SSP2 assumes medium fertility, medium mortality, medium migration, medium education, and central urbanization (i.e., the mean urbanization growth rate of historical experience for a certain country); SSP3 assumes high fertility, high mortality, low migration, low education, and slow urbanization; SSP4 assumes high fertility, high mortality, medium migration, polarized education (i.e., a certain group with very high education and large groups with low education), and fast urbanization; and SSP5 assumes low fertility, low mortality, high migration, high education, and fast urbanization [42,43].

Chinese population size projections between 2010 and 2050 for all ages under the 5 SSPs at 0.125° × 0.125° resolution were extracted from the global projections [44]. City-level population projections were then calculated by summing the populations of each grid cell that fell within a certain city boundary. Chinese population age structure changes between 2010 and 2050 under the 5 SSPs were obtained from the SSP Database (https://tntcat.iiasa.ac.at/SspDb).

### Ozone–mortality concentration–response functions (CRFs) and baseline mortality

Because China has higher air pollution levels and may also differ in terms of age structure, population sensitivity to air pollution, and components of air pollution mixture compared to developed countries [45], we used China-specific ozone CRFs in the present study. Accordingly, we applied pooled estimates of the association between short-term exposure to MDA8 ozone and daily mortality from a recent nationwide study in 272 Chinese cities [46] (Table 1). The nationwide time-series study was conducted between 2013 and 2015, during which daily air pollution concentrations and mortality counts were collected by the China National Environmental Monitoring Center and the China Disease Surveillance Points system, respectively [46]. In that study, a 2-stage approach was used to obtain national average associations between ozone concentration and cause-specific (cardiovascular, respiratory, or other), age-group-specific (5–64 years, 65–74 years, and ≥75 years), and seasonal mortality for all non-accidental deaths. Briefly, generalized linear models were first applied to estimate the acute ozone effects on mortality in each city, and then hierarchical Bayesian models were used to pool the city-specific estimates. Daily deaths for children under 5 years were too few and thus were excluded in that nationwide time-series study [46]. Since the number of city-level daily non-accidental deaths for individuals aged 5–64 years was small (Table 1), no further divisions of this age group were applied [46]. No threshold below which no adverse health effects occur was assumed for the CRFs, as there is no evidence that supports a threshold in the relationship between short-term ozone exposure and mortality [27,47,48].

**Table 1:** Ozone–mortality concentration–response functions (CRFs) and summary statistics of daily mortality in 104 Chinese cities during 2013–2015.

Mortality	CRF^a^	Number of deaths			
		Mean ± SD	Minimum	Median	Maximum
*Cause of death*					
All non-accidental causes	0.24 (0.13, 0.35)	23 ± 23	3	18	165
Cardiovascular	0.27 (0.10, 0.44)	11 ± 10	1	9	65
Respiratory	0.18 (−0.11, 0.47)	3 ± 4	0	2	34
*Age-group-specific non-accidental deaths*					
5–64 years	0.13 (−0.23, 0.48)	5 ± 5	1	4	43
65–74 years	0.19 (0.03, 0.34)	5 ± 5	1	4	37
≥75 years	0.42 (0.21, 0.64)	13 ± 13	1	9	84
*Seasonal non-accidental deaths*					
Warm (May–Oct)	0.20 (0.08, 0.31)	21 ± 21	3	15	155
Cold (Nov–Apr)	0.43 (0.21, 0.65)	20 ± 17	3	16	118

^a^CRFs are expressed as the percentage increase (95% confidence interval) in daily mortality associated with a 10-μg/m^3^ (approximately 5 parts per billion) increase of maximum daily 8-hour average ozone exposure on the current day and previous 3 days. CRFs were obtained from a previous nationwide time-series study in 272 Chinese cities [46].

City-level baseline cause-specific, age-group-specific, and seasonal mortality counts were also obtained from the previous publication [46]. Future changes in age-group-specific mortality rates and their 95% probability intervals (PIs) in 2050–2055 versus 2010–2015 were obtained using data from the United Nation’s 2017 World Population Prospects [49] and the MortCast package, which projects age-specific mortality rates using the Kannisto, Lee–Carter, and related methods as described in Ševčíková et al. [50]. The calculated changing ratios of mortality rates in 2050–2055 versus 2010–2015 in China were 0.68 (95% PI: 0.35–1.02) for individuals aged 5–64 years, 0.50 (95% PI: 0.26–0.73) for individuals aged 65–74 years, and 0.83 (95% PI: 0.61–1.05) for individuals aged ≥75 years. Because no projection data for city-level cause-specific and seasonal mortality rates were available in China, we assumed constant daily mortality counts for each city in the future when estimating future cause-specific and seasonal ozone-related acute excess mortality.

### Health impact assessment

We used the attributable fraction (AF) method to estimate the daily ozone-related acute excess mortality in 2013–2015 and 2053–2055, respectively. The AF is the fraction of baseline mortality attributable to ozone exposure, which is defined as [13,51]

$$AF=1-{exp}^{-\beta \times C}$$ (1)

where β is the coefficient of the CRF for ozone and *C* is the city-level daily ozone concentration (ppb) in each period. The city-level attributable daily deaths (ADD) for ozone exposure in each period was then calculated as follows:

$$ADD=Y_{b}\times POP\times AF=Y_{b}\times POP\times (1-e^{-\beta \times C})$$ (2)

where ADD represents the estimated city-level number of daily deaths attributable to ozone exposure in each period, *Y_b_* is the city-level baseline daily mortality rate, and POP is the city-level annual population in each period (i.e., population in 2010 for the historical period and population in 2050 for the future period). We then calculated the annual attributable deaths by summing ADD throughout the year. Finally, we estimated the effects of changes in ozone concentrations on mortality for each city by computing the changes in future annual attributable deaths in 2053–2055 relative to the historical annual attributable deaths in 2013–2015 (excess mortality).

We calculated the cause-specific, age-group-specific, and season-specific ozone-related acute excess mortality separately by applying corresponding CRFs and baseline mortality. We computed the GFDL-CM3 ensemble-averaged total excess mortality in 104 cities by combinations of RCPs and population scenarios. We used Monte Carlo simulations to quantify the uncertainty in our mortality estimates by incorporating uncertainty from coefficients of CRFs and model variability across 3 ensemble members of GFDL-CM3. We obtained the empirical confidence intervals (eCIs) from the empirical distribution across 1,000 coefficient samples and 3 ensemble members, assuming normal distributions for the estimated cause-specific, age-group-specific, and season-specific coefficients, respectively. When estimating future ozone-related acute excess mortality under population aging scenarios, we further incorporated the uncertainty from age-group-specific mortality rate changes in the Monte Carlo simulations by assuming normal distributions for the age-group-specific changes in total mortality rate separately. We did not include uncertainty associated with population projections in mortality estimates, because these are not reported in SSP population projections and our goal was scenario-based projections for understanding the implications of health consequences under future demographic and climatic conditions. To evaluate the influence of the fine-scale ozone projections on health impact assessments, we conducted a sensitivity analysis by directly using the coarse-scale bias-corrected ozone projections to estimate future ozone-related cause-specific excess mortality under a no population change scenario.

Four factors—ozone concentration changes due to climate and emission change, population size changes, population aging, and age-group-specific mortality rate changes—contribute to the total future ozone-related acute excess mortality. To decompose changes in ozone-related acute excess mortality, we estimated the contribution from each factor incrementally for the population aged 5 years and above with age-group-specific risk estimates. We calculated the effect due to climate and emission change by applying the no population change scenario, the effect due to population size by subtracting the climate and emission change effect from the effect when applying the population size changes under the 5 SSPs, the effect due to population aging by subtracting the effect when applying the population size changes from the effect when applying the population changes in both size and age structure under the 5 SSPs, and the effect due to age-group-specific mortality rate by subtracting the effect when applying population changes in size and age structure from the effect when applying both population changes in size and age structure and age-group-specific mortality rate changes.

## Results

### Historical and future ozone concentrations

During 2013–2015, the mean annual average of observed ozone concentrations in 104 Chinese cities was 41.6 ppb, with higher ozone concentrations observed in cities located along the east coast of China (Fig 1A). Ensemble mean projected changes in ozone in 2053–2055 relative to 2013–2015 differed by RCP (Fig 1B). Under the moderate climate and emission change scenario, RCP4.5, the mean annual average ozone concentration declined to 32.3 ppb in 2053–5055 relative to 2013–2015, reflecting reduction of O_3_ precursor emissions (S2 Fig). Under the high climate and emission change scenario, RCP8.5, the mean annual average ozone concentration increased to 46.9 ppb in 2053–5055 relative to 2013–2015. Of all days in the historical period, an average of 39.0% had ozone levels exceeding the WHO air quality guidelines (100 μg/m^3^, roughly equivalent to 50 ppb). In 2053–2055, the percentage of days with ozone concentrations exceeding the air quality guidelines declined to 10.0% under RCP4.5 but increased to 42.2% under RCP8.5. City-specific estimates of ozone changes indicated spatial heterogeneity, with larger decreases in southern cities under RCP4.5 and larger increases in northern cities under RCP8.5 (S3 Fig).

### Population size changes and population aging

Under the SSPs, population size in 104 Chinese cities would first increase from 2010 to 2030, then decrease from 2030 to 2050 (Fig 1C). The decrease in total population in 104 Chinese cities in 2050 relative to 2010 would range from −0.5% under SSP3 to −5.6% under SSP4. All the SSPs projected an aging Chinese population (Fig 1D). Compared with 2010, the proportions of the population aged between 65 and 74 years and 75 years and above in 2050 increased from 5.2% to 11.7%–14.0% and from 3.0% to 12.0%–19.0%, respectively.

### Ozone-related acute excess mortality under global climate and emission change

**Fig 2:**
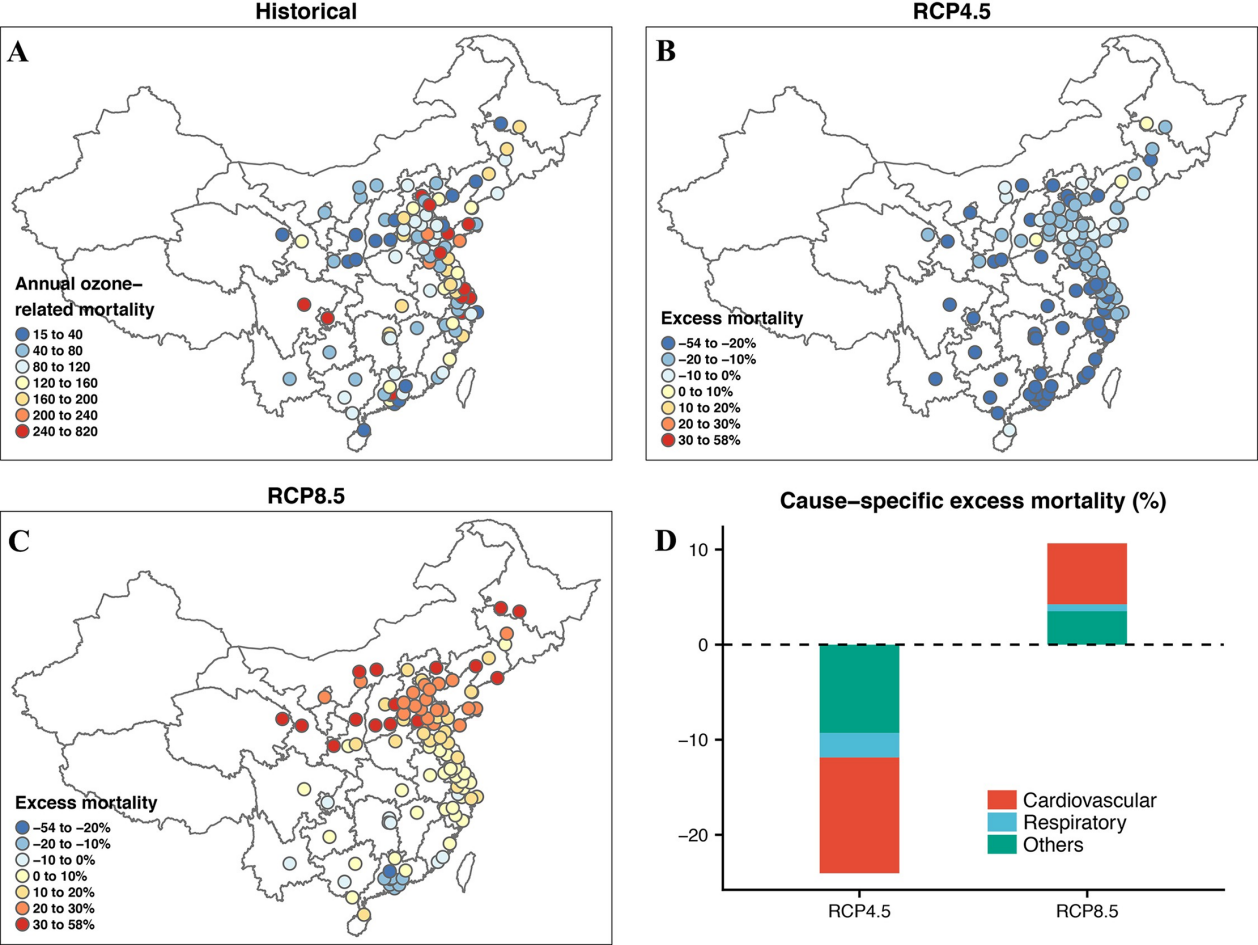
Impact of climate and emission change on ozone-related acute excess mortality. (A) Spatial distribution of historical annual ozone-related mortality in 104 Chinese cities during 2013–2015. (B) Spatial distribution of future changes (%) in annual ozone-related mortality under the RCP4.5 scenario in 104 Chinese cities during 2053–2055 relative to the historical period 2013–2015. (C) Same as (B) but under RCP8.5. (D) Future changes (%) in ozone-related mortality by cause of death (cardiovascular, respiratory, and other causes of non-accidental deaths) under RCP4.5 and RCP8.5. RCP4.5 and RCP8.5 represent moderate and high global warming and emission scenarios, respectively.

In the historical period, short-term exposure to ambient ozone contributed to a total of 13,856 non-accidental deaths annually in 104 Chinese cities. Historical ozone-related acute excess mortality varied substantially among cities, with the smallest attributable deaths in Haikou (15) and the largest attributable deaths in Nantong (820) (Fig 2A). Fig 2B shows that under a no population change scenario, decreasing mortality impacts in 2053–2055 relative to 2013–2015 under RCP4.5 occurred in most cities, with the largest decreases in southern cities. Under RCP8.5, larger increases in mortality occurred in northern cities whereas smaller increases or even decreases occurred in southern cities (Fig 2C). These spatial differences in ozone-related acute excess mortality reflected the spatial heterogeneity of future ozone changes under climate and emission change (S3 Fig). Relative to the historical period, annual ozone-related non-accidental mortality in 2053–2055 decreased by −3,332 (95% eCI: −5,877 to −2,191) deaths (−24.0%) under RCP4.5, but increased by 1,476 (95% eCI: 898 to 2,977) deaths (10.7%) under RCP8.5 (Table 2 and Fig 2D). Mortality from cardiovascular disease accounted for more than half of ozone-related excess deaths from global change (50.6% under RCP4.5 and 60.1% under RCP8.5), whereas mortality from respiratory disease accounted for only 10.7% under RCP4.5 and 6.9% under RCP8.5.

**Table 2:** Ensemble mean changes of annual ozone-related mortality in 104 Chinese cities in 2053–2055 relative to 2013–2015 under different climate and population scenarios.

Population	Population scenario	Baseline mortality rate	Cause of death	Mean change in mortality (95% empirical CI) by climate and emission change scenario	
				RCP4.5	RCP8.5
All ages^a^	No change	No change	All non-accidental causes	−3,332 (−5,877, −2,191)	1,476 (898, 2,977)
			Cardiovascular	−1,687 (−3,370, −776)	888 (404, 1,925)
			Respiratory	−357 (−1,138, 308)	101 (−75, 380)
Population aged 5 years and above^b^	SSP1	Age-group-specific mortality changes	All non-accidental causes	47,804 (20,602, 80,463)	78,560 (35,611, 131,541)
	SSP2		All non-accidental causes	35,004 (14,606, 60,544)	60,140 (26,299, 99,484)
	SSP3		All non-accidental causes	23,838 (8,609, 43,114)	44,063 (18,530, 73,377)
	SSP4		All non-accidental causes	36,357 (15,368, 63,214)	62,049 (27,497, 10,2124)
	SSP5		All non-accidental causes	47,765 (19,933, 80,981)	78,505 (34,083, 129,084)

^a^For the population including all ages, the calculation was based on the risk estimate of ozone-related mortality and annual baseline mortality for the whole population.

^b^For the population aged 5 years and above, the calculation was based on the risk estimates of ozone-related mortality and annual baseline mortality for the population age groups 5–64, 65–74, and 75+ years.

Using the coarse-scale (2.0° × 2.5°) ozone projections yielded larger ozone concentration changes in the future period, with an average of +1.3 ppb under RCP4.5 and +1.4 ppb under RCP8.5 in 104 Chinese cities (S4 Fig). Compared with using the fine-scale (0.25° × 0.25°) ozone projections, ozone-related acute excess mortality estimates were +36.6% greater under RCP8.5 and −16.3% smaller under RCP4.5. Biases due to coarse resolution presented large spatial variations across the 104 cities. These findings suggest that the ozone-related acute excess mortality in the 104 Chinese cities was sensitive to the spatial resolution of the ozone projections.

### Ozone-related acute excess mortality under both climate and population change

Using age-group-specific CRFs and baseline mortality yielded a larger ozone-attributed death burden (21,592) than using all-age CRFs and baseline mortality (13,856) in the historical period. Higher estimates of annual ozone-related acute excess mortality were observed when the population changed in both size and age structure under the 5 SSPs (Table 2). The evolving patterns of future ozone-related acute excess mortality under climate change differed in the 5 SSPs. The highest estimates were found using the SSP1 population scenario, whereas the lowest estimates were observed using the SSP3 population scenario.

**Fig 3:**
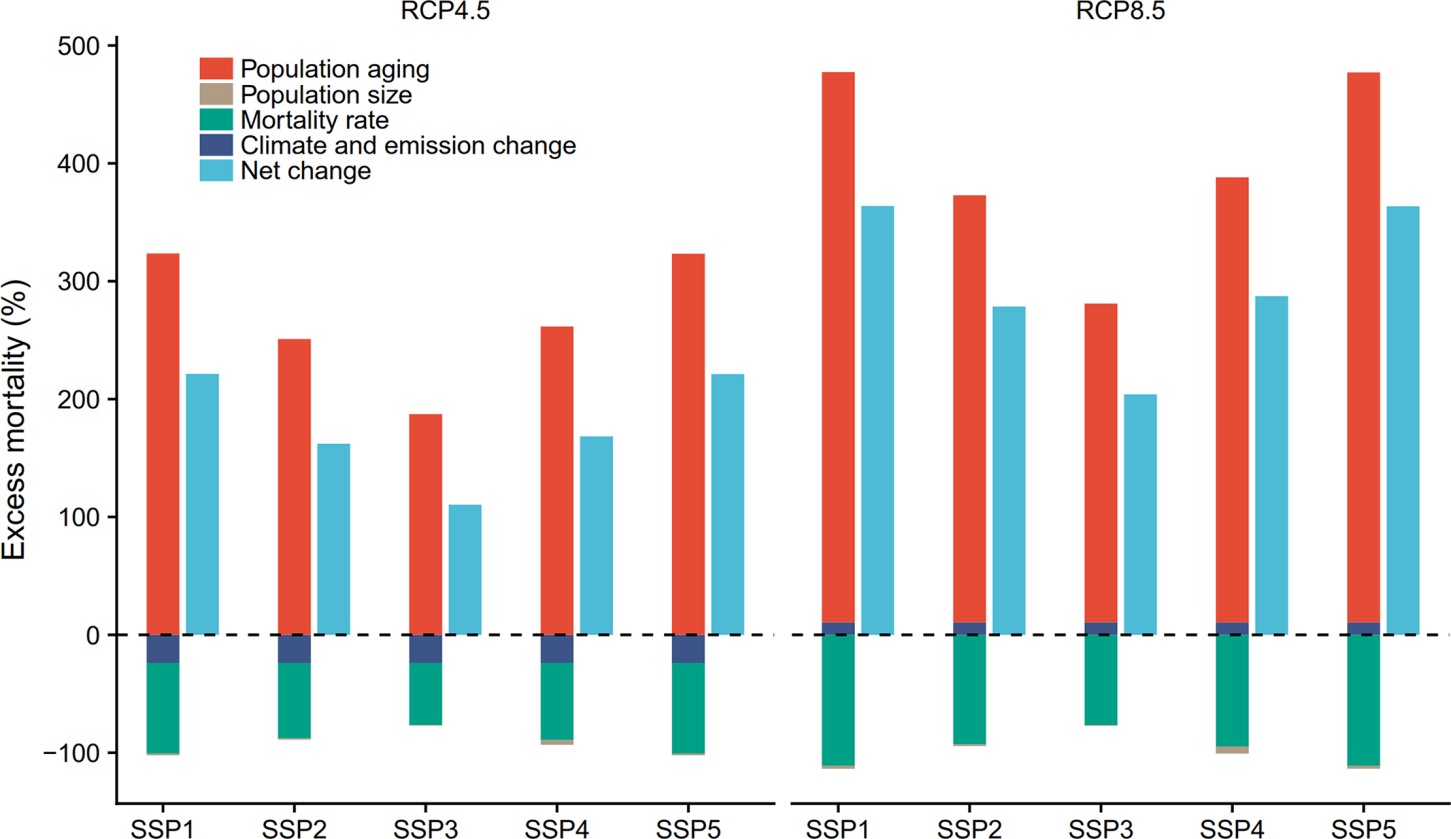
Changes in ozone-related mortality according to climate and population changes from 2013–2015 to 2053–2055. Population changes include both population size changes and population aging. Mortality rate indicates age-group-specific baseline mortality rate changes. Future changes (%) of annual ozone-related mortality for the population aged 5 years and above in 2053–2055 were calculated relative to the historical period 2013–2015. RCP4.5 and RCP8.5 represent moderate and high global warming and emission scenarios, respectively. SSP1–5 represent 5 population change scenarios under different shared socioeconomic pathways.

The evolving patterns of future ozone-related acute excess mortality reflected the influence not only of climate and emission change, but also of changes in population size, aging, and age-group-specific mortality rates. Fig 3 shows the contributions of these 4 factors to changes in ozone-related mortality from 2013–2015 to 2053–2055. Decreasing excess deaths owing to acute ozone exposure under RCP4.5 and age-group-specific mortality rate reductions were offset by population aging. Population size slightly declined in 2050 compared with 2010 (−5.6% to −0.5%), whereas the proportion of older people between 65 and 74 years and 75 years and above increased by 2–3 and 4–6 times from 2010 to 2050, respectively. Since the baseline mortality and CRFs for older people aged 75 years and above are 2.6 and 3.2 times greater, respectively, than for young people aged 5–64 years (Table 1), population aging alone results in 2–5 times greater ozone-related acute excess mortality under climate change (Fig 3). This large increase due to population aging fully offsets the decreases due to decreasing age-group-specific mortality rates (−111% to −53%). Consequently, net increases (110% to 221%) were noted under RCP4.5 for the 5 SSP population scenarios. Under RCP8.5, increases in ozone exposure from global climate and emission change combined with increases in population aging resulted in large net increases in ozone-related acute excess mortality (204% to 363%). Increases in population aging offset reductions in population size and age-group-specific mortality rates and dominated the net increases under RCP8.5.

### Seasonal variation of future ozone concentration and ozone-related acute excess mortality

**Fig 4:**
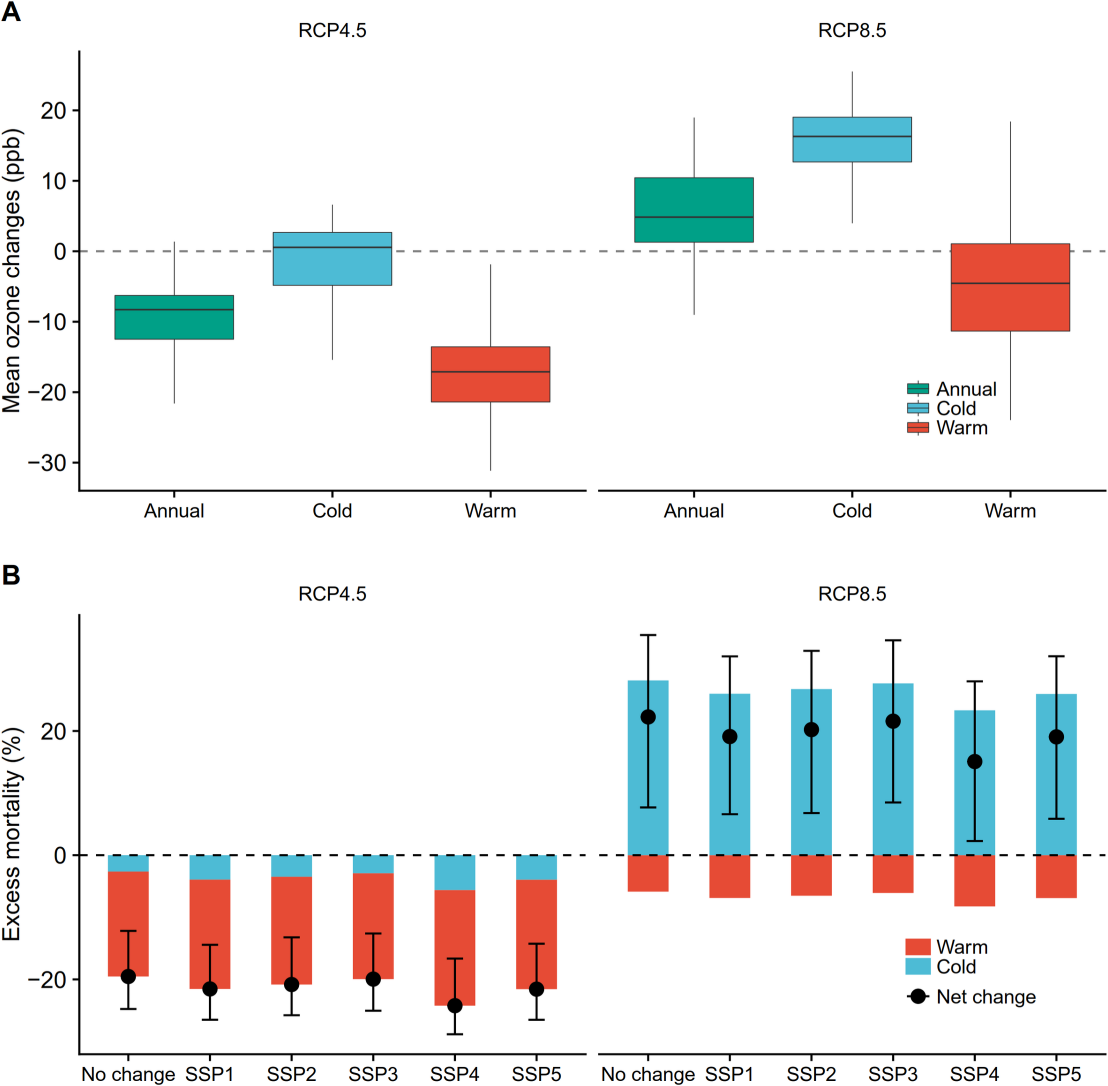
Seasonal changes in future ozone concentration and ozone-related mortality under climate and emission changes and population size changes. (A) Future changes in annual average daily ozone concentrations (parts per billion [ppb]) during the whole year, the warm season (May–October), and the cold season (November–April) in 2053–2055 relative to 2013–2015. RCP4.5 and RCP8.5 represent moderate and high global warming and emission scenarios, respectively. The horizontal line within each box represents the median change in ozone concentration among 104 cities, the lower and upper boundaries of the box indicate the 25th and 75th percentiles, and the ends of the whisker lines indicate the maximum and minimum concentrations within 1.5 times the interquartile range from the upper and lower box boundaries. (B) Future changes (%) in annual ozone-related mortality during the warm season and the cold season in 2053–2055 relative to 2013–2015. Population size scenarios include a no change scenario and 5 population change scenarios under shared socioeconomic pathways (SSPs). Vertical lines denote 95% empirical CIs of net change.

Seasonal ozone changes projected under both RCPs are reported in Fig 4A. Larger reductions in ozone concentrations were observed in the warm season (−16.6 ppb) than in the cold season (−1.7 ppb) under RCP4.5. An opposite pattern of attenuation in the warm season (−4.2 ppb) and a rise in the cold season (15.1 ppb) was noted under RCP8.5. Consequently, under RCP8.5, more ozone-related acute excess mortality was projected to occur in 2053–2055 in the cold season, whereas less warm season excess mortality was projected compared to 2013–2015. Under RCP8.5, increased ozone-related mortality in the cold season offset reductions in the warm season, leading to a net increase (22.3% [95% eCI: 7.7% to 35.4%]). In contrast, under RCP4.5, ozone-related acute excess mortality was projected to experience a net decrease (−19.5% [95% eCI: −24.8% to −12.2%]), which was mainly driven by the large reduction in warm season impacts. The seasonal estimates of ozone-related acute excess mortality remained similar when we considered population size changes under the 5 SSPs.

## Discussion

In this study, we projected future ozone-related acute excess mortality in 104 Chinese cities using statistically downscaled ozone projections at a fine spatial resolution (0.25° × 0.25°). Future changes in ozone concentration and related non-accidental deaths from 2013–2015 to 2053–2055 exhibited different patterns in China under RCP4.5 and RCP8.5. Due to the impact of global climate and emission change, an increase in ozone-related deaths was estimated under RCP8.5, whereas a decrease was noted under RCP4.5. Cardiovascular mortality accounted for more than half of the mortality impacts from global climate and emission change. Population aging will likely amplify the ozone-related acute excess mortality burden under both RCPs. Furthermore, under RCP8.5, increased ozone concentrations (+15.1 ppb) in colder months (November to April) will lead to more ozone-related deaths (28.1%) than those avoided (−5.9%) due to decreased ozone concentrations (−4.2 ppb) in warmer months. Thus, deteriorating ozone air quality in colder months will make a growing contribution to the adverse health impacts of climate change in China.

Our estimates for projected changes in annual average ozone concentration in 104 Chinese cities from 2013–2015 to 2053–2055 (−9.3 ppb under RCP4.5 and +5.3 ppb under RCP8.5) are broadly consistent with recent modeling studies based on the latest RCP scenarios, which reported a decreasing ozone concentration for RCP4.5 (around −7 to −6 ppb) and an increasing concentration for RCP8.5 (0–3 ppb) in China over 2000–2050 [52,53]. The decline of ozone concentrations under RCP4.5 is mainly due to the assumed large reductions in emissions of ozone precursors such as nitrogen oxides (NO_x_) and nonmethane volatile organic compounds (S2 Fig). Very few studies have evaluated future changes in ozone-related acute excess mortality under climate change in China [15–17]. It is difficult to make direct comparisons between estimates from this study and previous studies, as different climate and emission change scenarios, model simulations, baseline times, ozone metrics, causes of mortality, population size scenarios, CRFs, and study areas were applied. In general, without considering population change, our finding of increasing ozone-related mortality in 104 Chinese cities under RCP8.5 from 2013–2015 to 2053–2055 (10.7%) agrees with the 2005–2030 increase (approximately 20%) projected under a current legislation scenario in eastern China reported by a recent study [17]. We found more ozone-related deaths under RCP8.5 and fewer deaths under RCP4.5 in China in 2050 than in 2010, which is consistent with findings for East Asia in previous global studies [15,54].

Few studies have assessed the impacts of climate change on ozone-related cause-specific mortality, especially mortality from cardiovascular diseases. Constrained by available CRFs, previous studies focused on either respiratory mortality from long-term exposure [15,16] or total mortality from short-term exposure [9,10,13,17]. In this study, future ozone-related acute excess mortality from cardiovascular diseases was 5–8 times greater than that from respiratory diseases (Fig 2D). This was mainly due to the higher proportion of baseline cardiovascular mortality and the stronger associations of short-term ozone exposure with cardiovascular mortality [46]. In 2013, cardiovascular disease accounted for 44.6% of total non-accidental mortality in China, whereas respiratory disease only accounted for 12.8% [24]. Consistent with previous epidemiological studies [55–57], the ozone-related mortality risk estimates we obtained from a previous publication were larger for cardiovascular disease than for respiratory disease [46]. Our findings suggest that future interventions to reduce the burden of cardiovascular disease should also incorporate measures to reduce climate-change-induced ambient ozone pollution.

Future ozone-related acute excess mortality will be substantially amplified by an aging population in the 104 Chinese cities studied (Fig 3). Due to the higher baseline mortality rates in older people (Table 1) and the vulnerability of older people to ambient ozone pollution [22,23], population aging is projected to contribute 187%–324% and 270%–467% ozone-related acute excess mortality across the 5 SSPs under RCP4.5 and RCP8.5, respectively. The impact of population aging under SSP1 and SSP5 could offset the reduced deaths due to decreasing age-group-specific baseline mortality rates and lower ozone concentrations in 2053–2055 under RCP4.5. Although population size slightly decreases in 2050 relative to 2010 (Fig 1C), the steep increases in the elderly population lead to increasing ozone-related acute excess mortality attributable to population change. Our findings indicate that not considering population aging (i.e., using age-group-specific CRFs and baseline mortality rates) may greatly underestimate future ozone-related acute excess mortality.

The seasonal variations in ozone-related acute excess mortality burdens reflect changes in seasonal ozone concentrations (Fig 4A). Interestingly, a projected reversal of the ozone seasonal cycle (from a summer maximum to a winter and early spring maximum) under RCP8.5 in the 21st century was also found in urban southeastern China [29]. Opposing seasonal ozone changes (decreasing in summer but increasing in winter) have been identified in the US over a historical period with decreasing NO_x_ and volatile organic compound emissions (1998–2013) [58]. A recent study also found decreases in US summer ozone and increases in winter ozone under both RCP4.5 and RCP8.5, reflecting at least partially less ozone titration by NO_x_ in the cold season, with low chemical reactivity and declining NO_x_ emissions [59]. A recent study using a decadal record of satellite observations found that US cities were generally within a NO_x_-saturated regime in winter, thus decreasing NO_x_ emissions and a decreased NO_x_ titration effect could lead to increased wintertime ozone [60]. Under RCP8.5, the approximate doubling of global methane abundances yields higher ozone concentrations, particularly during the cold season, when the ozone lifetime is longer [29]. In this study, we found decreases in warm season ozone and a reversal of ozone seasonal cycle under both RCPs (Fig 4A). This may be due to declines in NO_x_ emissions in China (S2A Fig), which may largely control the shape of the ozone seasonal cycle [29]. A warmer climate would enhance the warm season ozone (climate change penalty); however, the NO_x_ emission reductions under both RCP4.5 and RCP8.5 can fully offset this climate change penalty, resulting in overall decreases in warm season ozone [29].

Few studies have evaluated the seasonal variations of acute ozone-related health impacts under climate change [9,11]. Consistent with our findings, ozone-related mortality projections were found to decrease during the warm season (May to September) and to increase during the cold season (October to April) under RCP8.5 in the US for 2057–2059 [9]. In contrast, most of the projected increases in ozone-related mortality were found in summer months (April to September) in Europe, when applying a threshold of 35 ppb [11]. The inconsistency of this finding with ours may be due to the no threshold assumption applied in the present study, which measures the excess deaths attributable to increases in ozone concentrations below 35 ppb during the winter season. As there is no clear evidence for the existence of a threshold for the relationship between short-term ozone exposure and daily mortality [27,47,48], applying a relatively high concentration threshold would significantly underestimate the health impact of climate change via changes in ambient ozone pollution. Most previous studies only projected ozone-related mortality in the warm season, which we emphasize might underestimate the adverse health effects of ozone exposure throughout the year [25]. A recent study found that applying a whole year ozone exposure metric with updated risk estimates led to more than double the estimated global long-term ozone-attributable respiratory mortality compared with using a warm season metric with lower risk estimates [61]. Chinese urban populations are still exposed to ambient ozone pollution in cold months, with an average of 181 minutes/day spent outdoors in winter [62]. In winter, urban populations in northeastern China spend only 90 minutes/day outdoors, whereas those in southern China spend 218 minutes/day outdoors [62]. Under a warming climate, outdoor exposure to ozone might change due to changing time-activity patterns, which could influence the seasonality of ozone-attributed mortality in the future. Moreover, consistent with the nationwide seasonal estimates applied in this study [46], most previous Chinese studies found that the mortality effects of short-term ozone exposure were higher in the cold season than in the warm season in Shanghai [63], Suzhou [31,64], Zhengzhou [65], Guangzhou [66,67], the Pearl River Delta [68], and Jiangsu Province [47]. Thus, future research projecting future ozone-related mortality under a changing climate in different seasons may be warranted.

Our results are based on a recent reference time period (2013–2015), which was chosen due to the availability of national measurements of ambient ozone concentrations. Compared with earlier time periods (e.g., 2000–2002), this reference time period has higher levels of ozone pollution [69] and greenhouse gas emissions [70] in China. Hence the estimates of ozone-related acute excess mortality under climate and emission change reported here are likely lower than those one would find using earlier reference time periods. Moreover, our estimates assume independent effects of ozone and fine particulate matter (PM_2.5_) on daily mortality as the CRF estimates we used here were robust after adjustment for co-exposure to PM_2.5_ in 2-pollutant time-series models [46]. Previous epidemiological studies also found evidence that PM_2.5_ is unlikely to confound the association between short-term ozone exposure and daily mortality [47,71]. Though focused on China, our findings, taken together with studies conducted in other regions [9–11,13] and worldwide [15], provide generally consistent evidence on increasing ozone-related mortality burden attributable to climate change under high emission scenarios.

To the best of our knowledge, this study is the first to account for population aging through age-group-specific risk estimates and for declining baseline age-group-specific mortality rates to understand future ozone-related mortality under alternative scenarios of climate and population change. This is also one of the first studies to apply season-specific risk estimates and baseline mortality rates to assess future mortality from changes in ambient ozone pollution attributable to climate change. The future ozone-related mortality burden in 104 Chinese cities is substantially reduced under RCP4.5, which includes effective emission mitigation strategies [35]. Our findings indicate that stricter mitigation measures will be needed in China to avoid the increasing health burden of climate change through poor ozone air quality.

There are several limitations in our study. First, only 1 global chemistry–climate model was applied to project future ozone concentrations. The choice of climate and air quality models has been found to dominate the uncertainty (approximately 48% to 97%) in projecting ozone-related health impacts [15,23]. In a recent multi-model study, future global ozone-attributed mortality estimates under RCP8.5 were found to be widely spread among models, while the atmospheric component of GFDL-CM3 produced estimates close to the multi-model averages [15]. Though 3 ensemble members were used to lessen uncertainty arising from internally generated climate variability to some extent, future multi-model studies (e.g., the Aerosol Chemistry Model Intercomparison Project [72]) will further address the uncertainty arising from global model projections. Second, the statistical downscaling applied in this study may result in an additional source of uncertainty irrespective of the nature of the global chemistry–climate model. Third, the effects of changing climate were not separated from the effects of changing ozone precursor emissions under RCP4.5 and RCP8.5 in this study. Fourth, the categories of age groups were chosen based on a previous nationwide time-series study [46], which was limited by the sample size of each age group and was unable to report the ozone–mortality CRFs for customary age groups such as 0–5 years, 6–14 years, and 15–65 years. Hence, ozone-related acute excess mortality was not estimated separately for children in our study. Children may spend more time outside playing and exercising, which can increase their vulnerability to the health impacts of ozone exposure [73]. Further investigation is needed to quantify the impacts of climate change on children’s health [74]. Fifth, the potential interaction between ozone and temperature on human health was not considered [64,75]. Finally, due to the scarcity of ozone–mortality CRF cohort studies in China, long-term effects of ozone on mortality were not included in the present study. Recent cohort studies in the US found that a 10-ppb increase in long-term ozone exposure is associated with a 1.1% to 2.0% increase in all-cause mortality [76,77], which is larger than the effect of short-term ozone exposure (e.g., a 0.52% increase in mortality per 10-ppb increase in ozone concentration in the US [55]). Although joint assessments of acute and chronic effects of ozone exposure on mortality are scarce, future investigations with available long-term CRF estimates in China may lead to larger estimated health impacts of climate change.

## Conclusion

Overall, we have shown that the future mortality burden for the Chinese population of short-term exposure to ambient ozone pollution will increase under plausible scenarios of climate and emission change and population aging. Population aging contributes substantially to the future ozone-related acute excess mortality. People with cardiovascular disease may require more care in a changing climate. Cold season ozone may play a more important role than currently recognized in future changes of ozone-related mortality in China. The differences in the direction and magnitude of future changes in ozone-related mortality attributable to climate and emission change under RCP4.5 and RCP8.5 suggest that climate change mitigation actions, such as preventing atmospheric methane from doubling, are needed to prevent a rising health burden from exposure to ambient ozone pollution in China.

## Disclaimer

The authors are solely responsible for the content of this paper and do not represent the official view of the EPA.

## Supporting information

S1 Fig Spatial distribution of mean maximum daily 8-hour average (MDA8) ozone concentration (ppb) in China during the period April 27, 2013, to October 31, 2015. A total of 778 national ambient ozone monitoring sites were involved in this study. Note that many sites overlap due to their proximity.
(TIF)

S2 Fig Changes of annual anthropogenic emission density used in the GFDL-CM3 model in China (18°~48° N, 100°~128° E) over 2000–2050 under RCP4.5 and RCP8.5 for (A) nitrogen oxides (NOx) (Tg N/year), (B) nonmethane volatile organic compounds (VOCs) (Tg C/year), and (C) carbon monoxide (Tg CO/year).
(TIF)

S3 Fig Spatial distribution of city-specific changes in annual average daily ozone concentration (ppb) under the RCP4.5 and RCP8.5 scenarios. (TIF)

S4 Fig Impact of using coarse resolution (2.0° × 2.5°) ozone projections on city-level ozone concentration changes and cause-specific excess mortality estimates. (A) Difference between mean ozone changes in 104 Chinese cities at coarse and fine resolution (0.25 × 0.25°). The horizontal line within each box represents the median concentration among 104 cities, the lower and upper boundaries of the box indicate the 25th and 75th percentiles, and the ends of the whisker lines indicate the maximum and minimum concentrations within 1.5 times the interquartile range from the upper and lower box boundaries. (B) Future changes (%) in ozone-related mortality by cause of death (cardiovascular, respiratory, and other causes of non-accidental deaths) based on coarse resolution ozone projections. RCP4.5 and RCP8.5 represent moderate and high global warming and emission scenarios, respectively. (C) Percent difference between cause-specific ozone-related acute excess mortality at coarse and fine resolutions. (D) Spatial distribution of percent difference between cause-specific ozone-related acute excess mortality at coarse and fine resolutions in 104 Chinese cities in 2053–2055 relative to 2013–2015 under RCP8.5. (E) Same as (D) but under RCP4.5.
(TIF)

S1 Method The statistical downscaling method: Bias-correction spatial disaggregation (BCSD). (PDF)

## Abbreviations

CRF: concentration–response function
eCI: empirical confidence interval
GFDL-CM3: Geophysical Fluid Dynamics Laboratory chemistry–climate model CM3
MDA8: maximum daily 8-hour average
NO_x_: nitrogen oxide(s)
ppb: parts per billion
PI: probability interval
RCP: Representative Concentration Pathway
SSP: shared socioeconomic pathway
