# Supplementary figures and images for: Future ozone-related acute excess mortality under climate and population change scenarios in China: A modeling study
Source: PLoS Med. 2018 Jul 3;15(7):e1002598. doi: 10.1371/journal.pmed.1002598 (PMC6029756; doi:10.1371/journal.pmed.1002598)

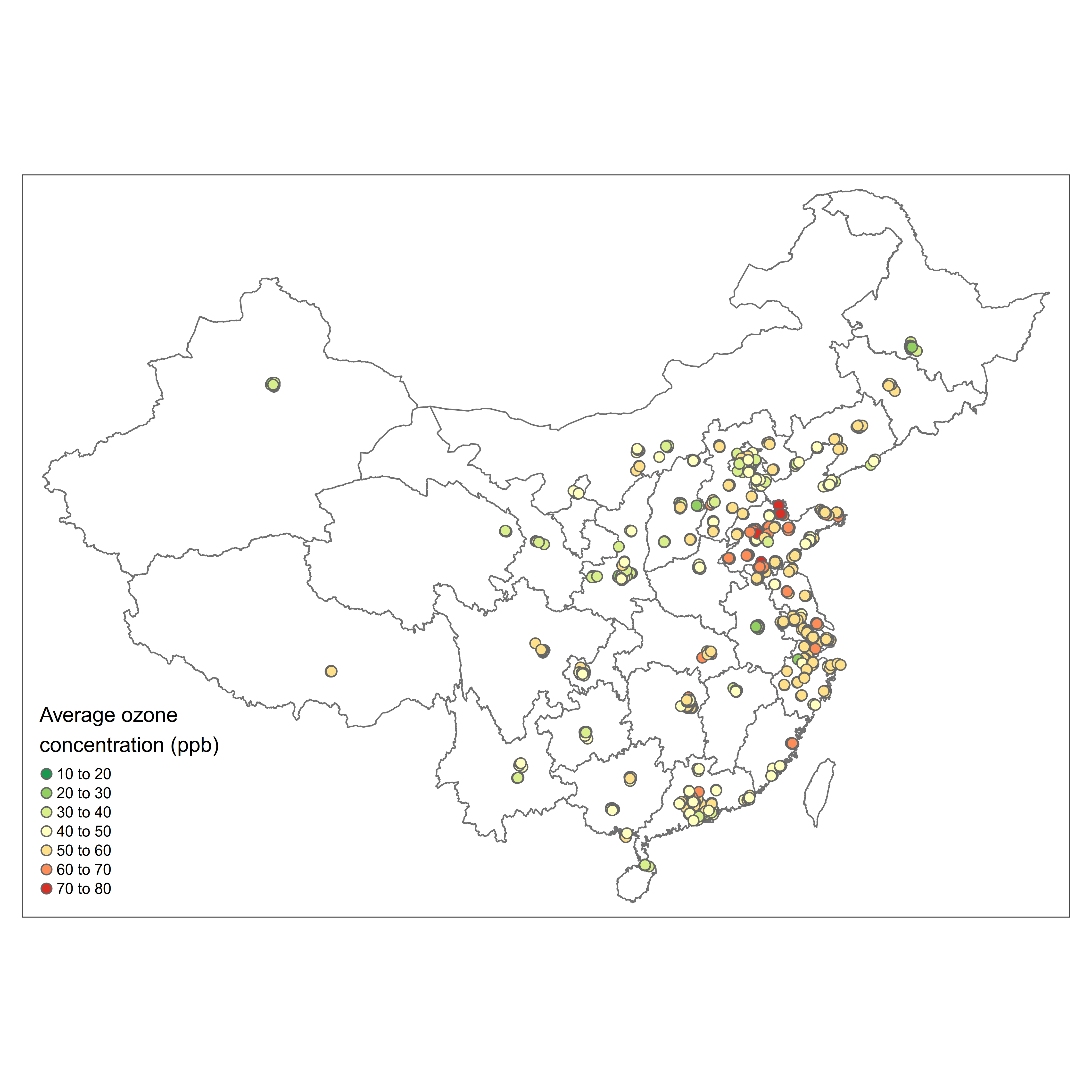

Supplement: S1 Fig — A total of 778 national ambient ozone monitoring sites were involved in this study. Note that many sites overlap due to their proximity. (TIF) [file pmed.1002598.s001.tif]

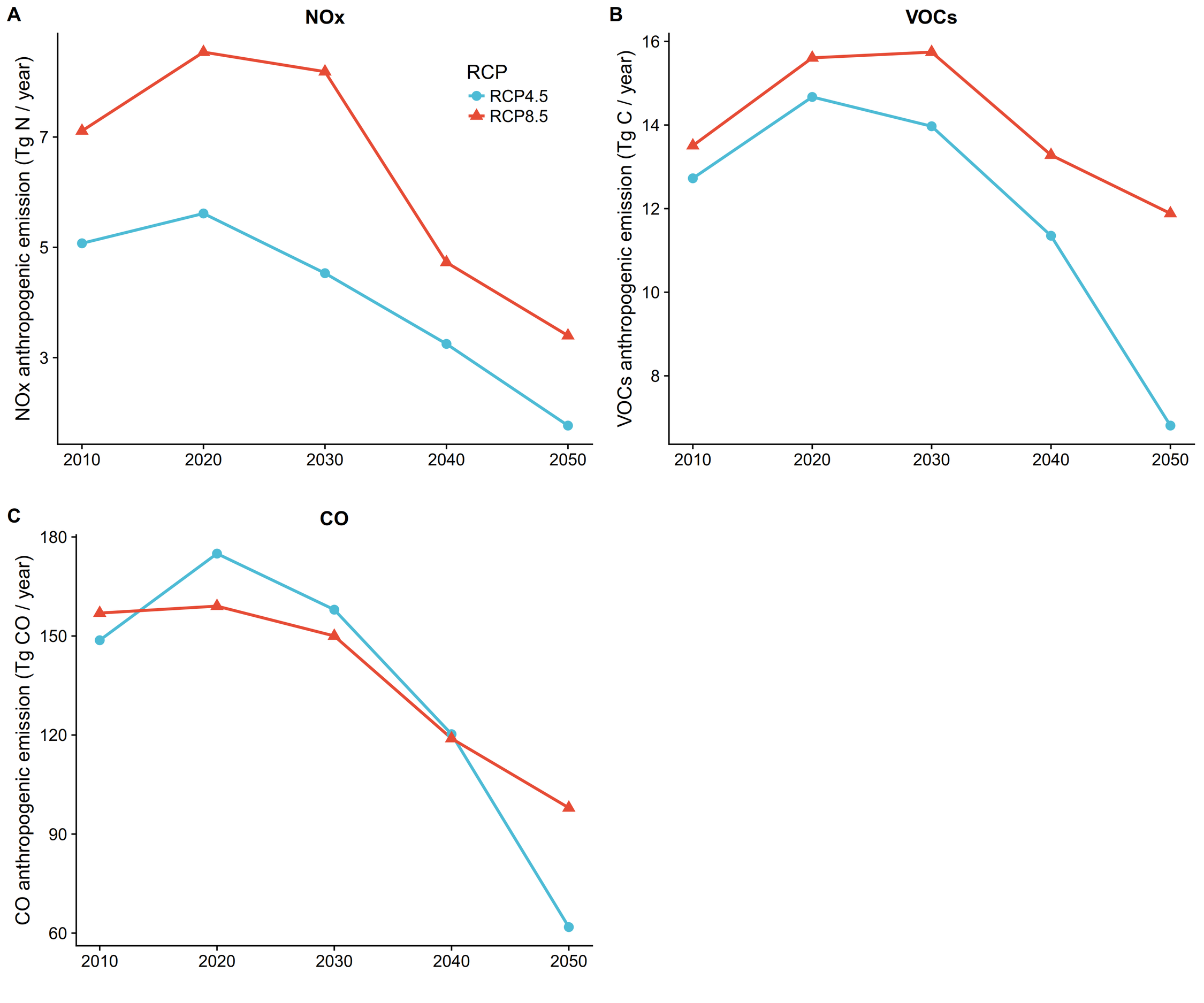

Supplement: S2 Fig — Changes of annual anthropogenic emission density used in the GFDL-CM3 model in China (18°~48° N, 100°~128° E) over 2000–2050 under RCP4.5 and RCP8.5 for (A) nitrogen oxides (NOx) (Tg N/year), (B) nonmethane volatile organic compounds (VOCs) (Tg C/year), and (C) carbon monoxide (Tg CO/year). (TIF) [file pmed.1002598.s002.tif]

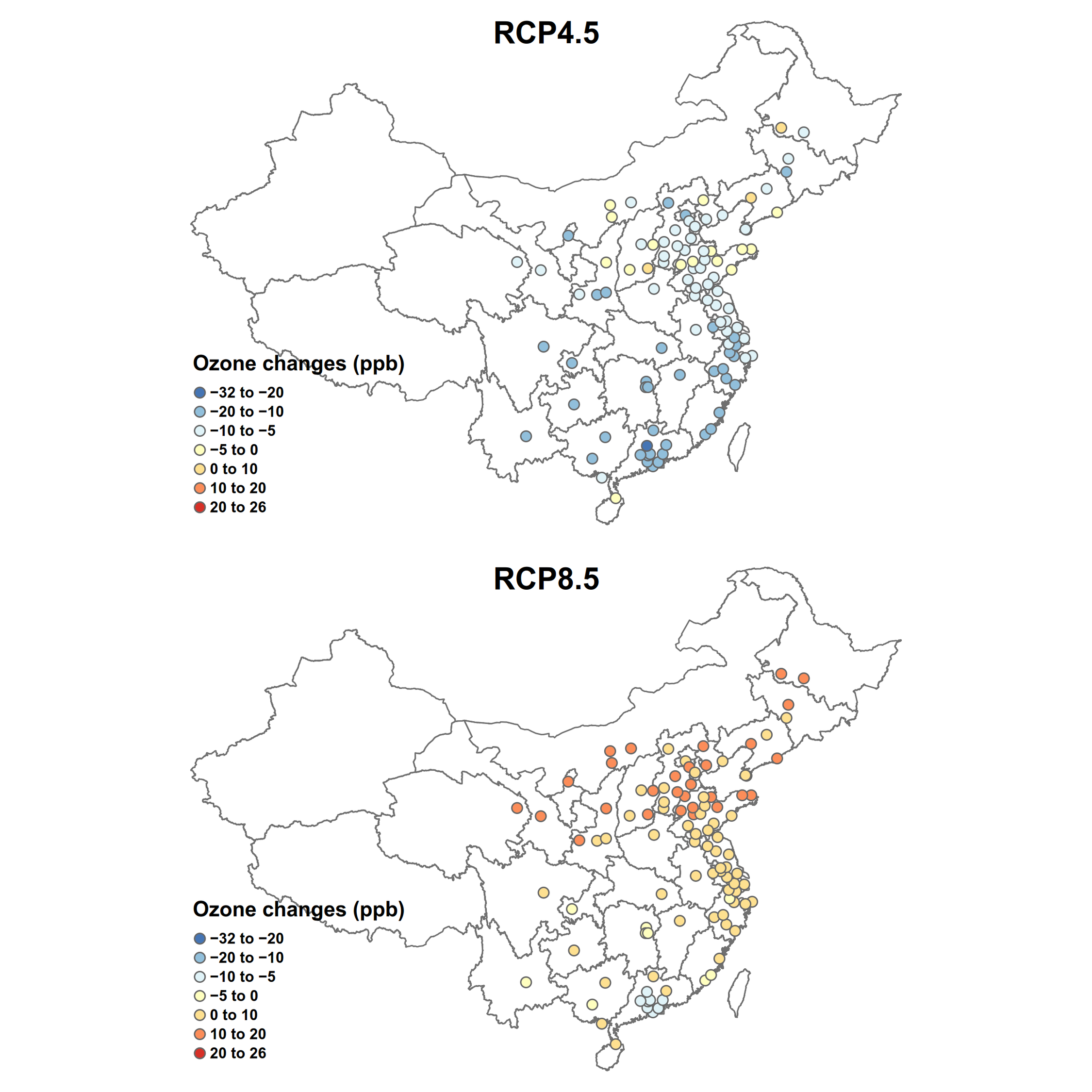

Supplement: S3 Fig — (TIF) [file pmed.1002598.s003.tif]

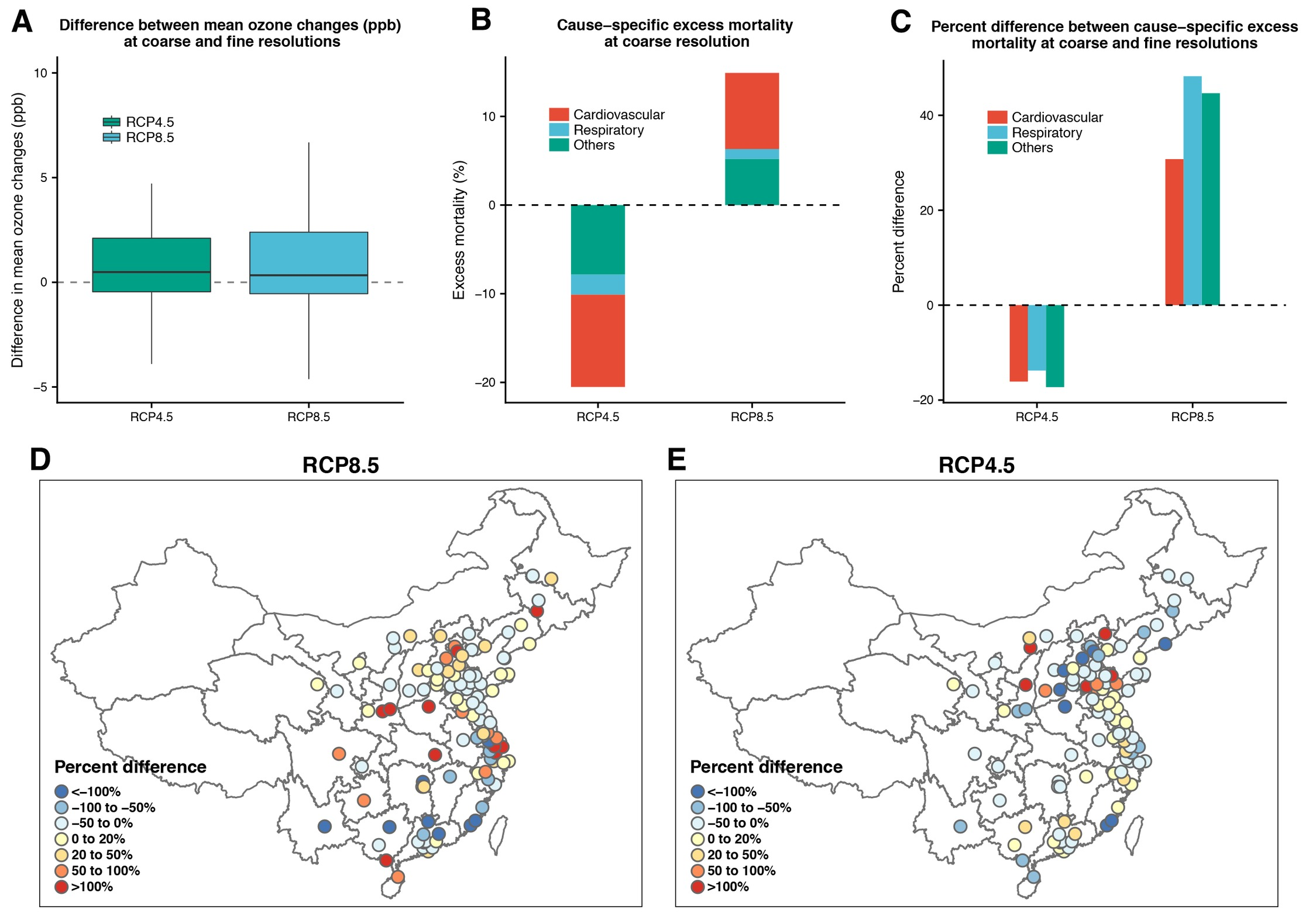

Supplement: S4 Fig — (A) Difference between mean ozone changes in 104 Chinese cities at coarse and fine resolution (0.25 × 0.25°). The horizontal line within each box represents the median concentration among 104 cities, the lower and upper boundaries of the box indicate the 25th and 75th percentiles, and the ends of the whisker lines indicate the maximum and minimum concentrations within 1.5 times the interquartile range from the upper and lower box boundaries. (B) Future changes (%) in ozone-related mortality by cause of death (cardiovascular, respiratory, and other causes of non-accidental deaths) based on coarse resolution ozone projections. RCP4.5 and RCP8.5 represent moderate and high global warming and emission scenarios, respectively. (C) Percent difference between cause-specific ozone-related acute excess mortality at coarse and fine resolutions. (D) Spatial distribution of percent difference between cause-specific ozone-related acute excess mortality at coarse and fine resolutions in 104 Chinese cities in 2053–2055 relative to 2013–2015 under RCP8.5. (E) Same as (D) but under RCP4.5. (TIF) [file pmed.1002598.s004.tif]
