# Supplementary material for: Future ozone-related acute excess mortality under climate and population change scenarios in China: A modeling study
Source: PLoS Med. 2018 Jul 3;15(7):e1002598. doi: 10.1371/journal.pmed.1002598 (PMC6029756; doi:10.1371/journal.pmed.1002598)
Supplement: S1 Method — (PDF) [file pmed.1002598.s005.pdf]

S1 Method. The statistical downscaling method: Bias-Correction Spatial Disaggregation (BCSD)

The detailed two-step procedure of the BCSD is described below.

(1) Bias-correction

In the bias-correction step, daily ozone observations (daily maximum 8-h average, MDA8) at 778 monitoring sites were first assigned to the fine-scale (0.25° × 0.25°) grid cells. The values of fine-scale observations were determined by the average value of monitoring sites within each grid cell. The fine-scale observations were then resampled to coarse-scale (2.0° × 2.5°, latitude × longitude) using a bilinear interpolation, which enables direct comparisons between observations and historical simulations of the Geophysical Fluid Dynamics Laboratory (GFDL) chemistry-climate model CM3 (GFDL-CM3). In a given 2.0° × 2.5° grid cell, for each of the 12 months, all daily values within the same month in a study period were used to construct a distribution function of daily values in this month. Biases were then identified by comparing the corresponding distribution functions between historical GFDL-CM3 simulations and observations. Assuming that the model bias remains the same in the future, we adjust the daily values in the future simulations by the identified bias for each ensemble member using a quantile-mapping approach. Note that the bias correction is pairing the daily observations with simulations within the same month, rather than pairing them day by day. This is because in a climate model, the modeled day isn't exactly the same as the observed meteorology on any given day.

Quantile mapping bias-correction (QmapBC) is a common approach to correct biased model simulations by finding a transfer function between model simulations and observations which maps the distribution of model simulations onto the distribution of observations [1]. Quantile mapping constructs cumulative distribution functions (CDFs) for both observations and historical model simulations to form a month- and grid-specific "quantile map", where biases are identified at each percentile. For each grid cell, CDFs were composed of all daily

values in each month (e.g., all daily values in August during the study period) to account for the monthly spatial pattern of ozone observations. Specifically, the basic idea of quantile mapping is to find the observational value X_o based on the assumption that:

$$F_o(X_o) = F_{mh}(X_{mh}) \quad [1]$$

where $F_o()$ is the CDF of observational value X_o; $F_{mh}()$ is the CDF of historical model simulations X_{mh}. This is equivalent to:

$$X_o = F_o^{-}(F_{mh}(X_{mh})) \quad [2]$$

Where $F_o^{-}()$ is the inverse $F_o()$. Assuming that this transfer function remains the same in the future, for the future model simulations X_{mf}, the bias-corrected value is $F_o^{-}(F_{mh}(X_{mf}))$.

To perform the quantile mapping, a common nonparametric transformation approach was used to obtain the CDFs of the observed and modeled MDA8 ozone values for regularly spaced quantiles with a 1% interval. The CDFs are estimated using empirical percentiles and values in between the percentiles are approximated using linear interpolation [1].

Compared with historical observations, GFDL-CM3 simulations during 2013-2015 were generally larger (+17 ppb) and had narrower probability density distributions (PDFs) under both RCP scenarios (Method Fig1). The standard deviation of ozone observations in 2013-2015 was higher than that of GFDL-CM3 simulations in RCP4.5 but similar to that of GFDL-CM3 simulations in RCP8.5.

Using the QmapBC method, the PDFs of bias-corrected GFDL-CM3 simulations were quite similar to that of historical observations under the RCP8.5 scenarios (Method Fig2). The PDFs of GFDL-CM3 simulations for all three ensemble members under the RCP4.5 scenario became wider (i.e., larger standard deviations) after the QmapBC correction.

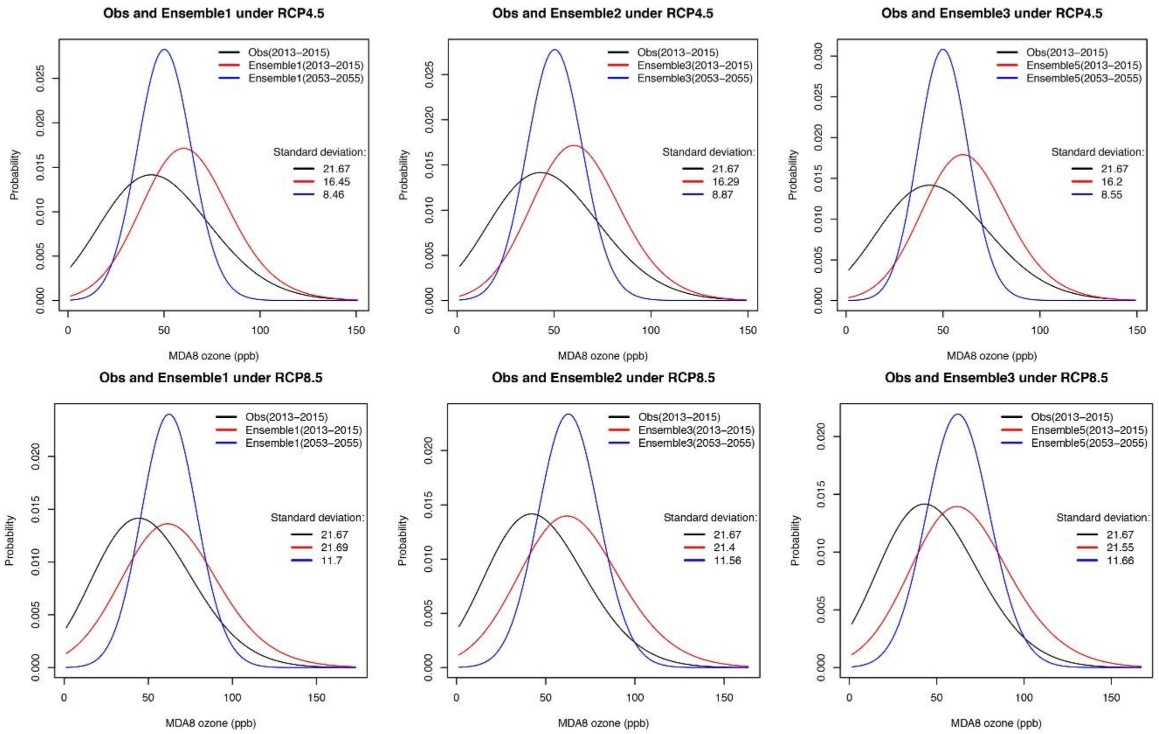

Method Fig1. Probability density function of MDA8 ozone at 2.5°×2.0° resolution for observations and ensemble members of GFDL CM3 simulations in different time periods under the RCP4.5 and RCP8.5 scenarios.

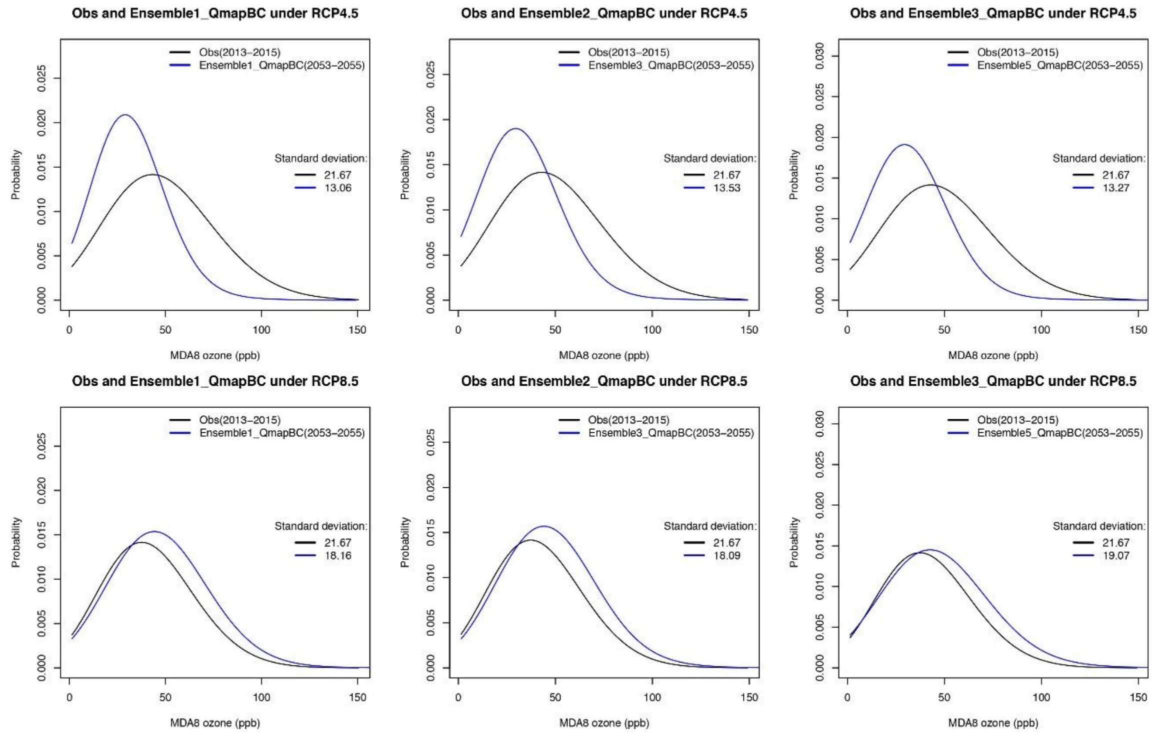

Method Fig2. Probability density function of MDA8 ozone at 2.5°×2.0° resolution for observations and ensemble members of bias-corrected GFDL CM3 simulations using the

quantile mapping method (QmapBC) in different time periods under the RCP4.5 and RCP8.5 scenarios.

The seasonal changes in 2053-2055 vs. 2013-2015 in GFDL-CM3 model ensembles were maintained in the bias-corrected simulations (Method Fig3). The patterns of monthly changes in mean ozone concentrations for GFDL-CM3 ensemble members are similar to the bias-corrected simulations under RCP4.5 and RCP8.5.

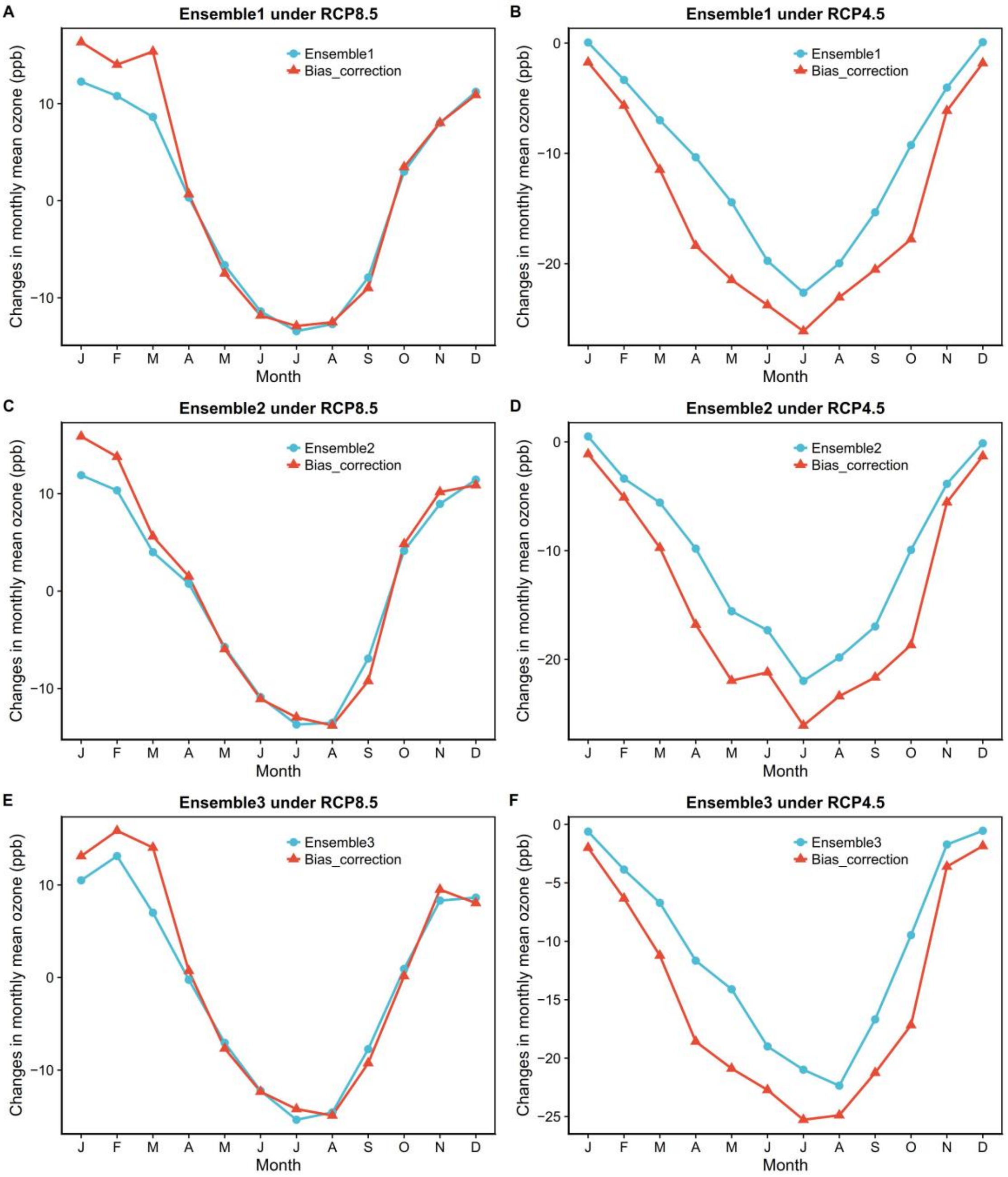

Method Fig3. Changes in monthly mean MDA8 ozone at 2.5°×2.0° resolution in 2053-2055 vs. 2013-2015 for ensemble members of GFDL CM3 simulations and bias-corrected simulations under the RCP4.5 and RCP8.5 scenarios.

(2) Spatial disaggregation

The spatial disaggregation spatially translates the bias-corrected future model simulations from the coarse-scale (2.5° × 2.0°) to the targeted fine-scale (0.25° × 0.25°). Similar to the concept of 'spatial climatology' used in the spatial disaggregation step of downscaling the CMIP5 climate projections in the U.S.A.[2], the spatial pattern of observed ozone was adopted to guide spatial disaggregation. In short, the spatial pattern of monthly mean observed ozone at 0.25° × 0.25° resolution was used as the targeted downscaled spatial pattern of monthly mean future simulations. The bias-corrected future model simulations were used to compute the time pattern (i.e., day-by-day) of changing factors for MDA8 ozone concentrations at each fine-scale grid. Then, the temporal scaling factors were added to the fine-scale spatial pattern to get the downscaled simulations. The steps of spatial disaggregation are as follows:

(1) Subtract the coarse-scale spatial patterns of observed monthly mean MDA8 ozone from the bias-corrected future daily projections to get the bias-corrected temporal scaling factors for each grid in the future period;

(2) Interpolate of temporal scaling factors from coarse-scale to targeted fine-scale using the bilinear interpolation method, which is based on geolocations and does not account for covariates such as landscape;

(3) Add the temporal scaling factors at fine-scale to the targeted fine-scale spatial patterns of monthly mean MDA8 ozone concentrations to get the final spatially downscaled future ozone simulations.

To illustrate each step of spatial disaggregation, CM3 Ensemble1 simulations on August 17, 2035 under RCP8.5 were used as an example in Method Fig4.

Spatial disaggregation of bias-corrected ozone

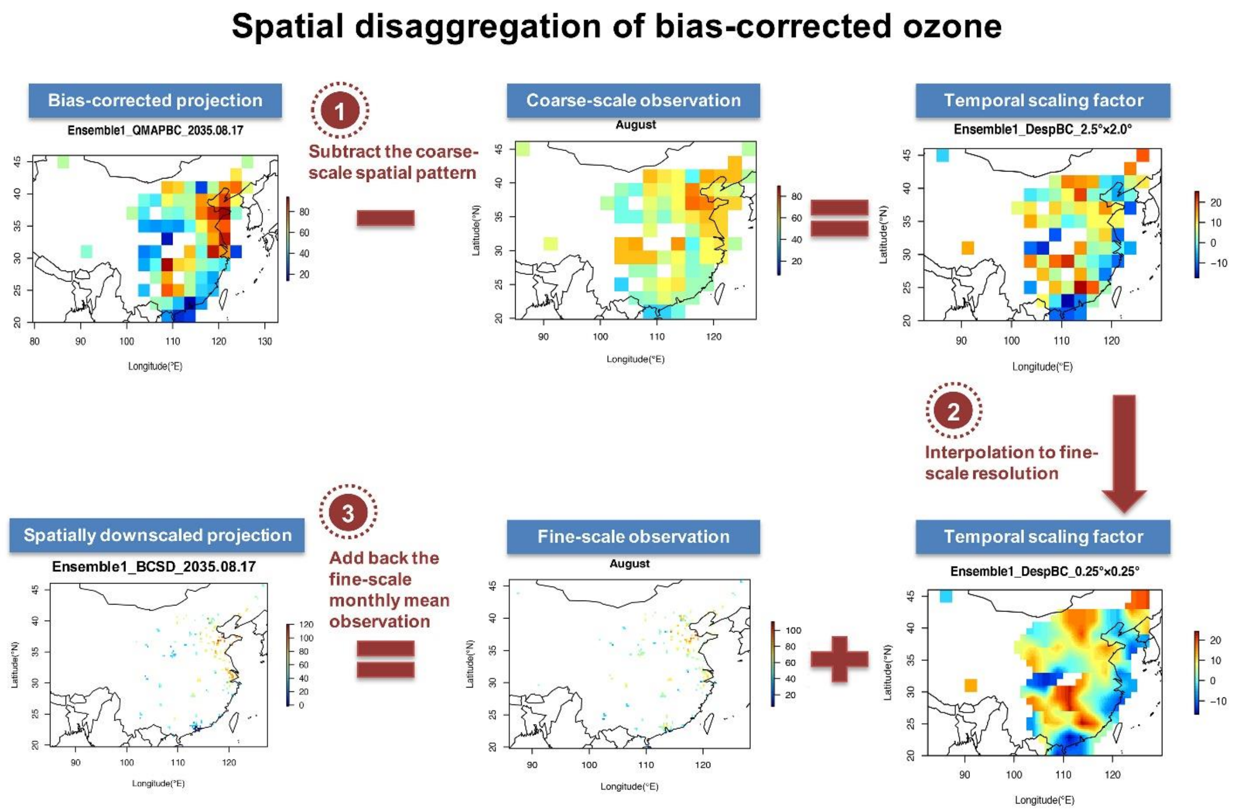

Method Fig4. Illustration of spatial disaggregation using Ensemble 1 simulations on August 17, 2035 as an example.

In the spatial disaggregation, the spatial pattern of monthly mean observation was applied as a spatial guide. Its coarse-scale spatial distribution was first subtracted from bias-corrected projections. Then its fine-scale spatial distribution was added back to the temporal scaling factor. Thus, the spatial distribution of observations were not kept the same as that in the future. Method Fig5 shows the spatial distribution of city-level annual mean ozone projections in 2053-2055 before (BC_2053-2055) and after (BCSD_2053-2055) spatial downscaling. The spatial distributions in 2053-2055 after spatial downscaling were generally similar to those before spatial downscaling (spearman's rank-order correlation coefficient: 0.81 under RCP4.5 and 0.85 under RCP8.5).

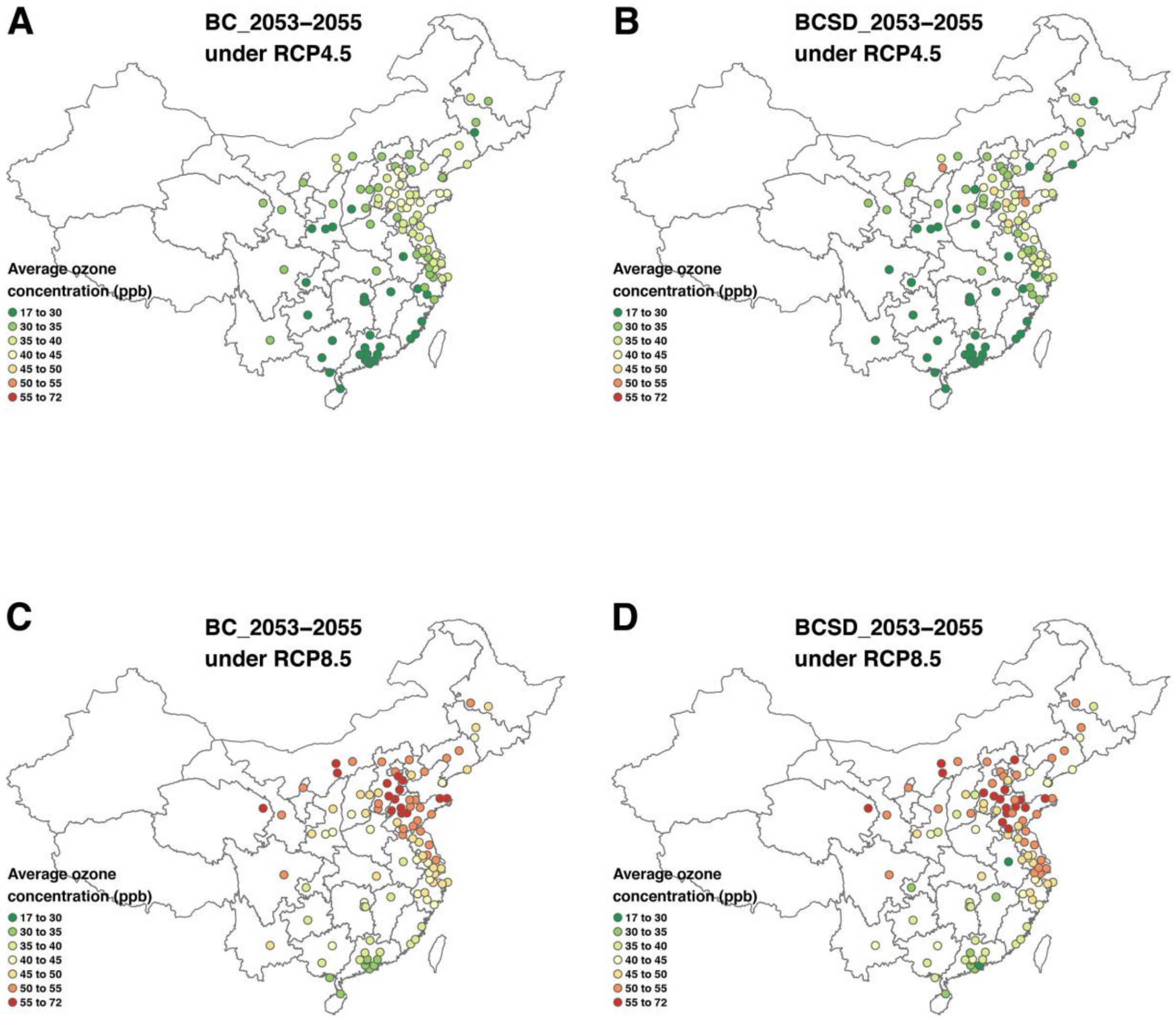

Method Fig5. Spatial distribution of annual average ozone projections in 2053-2055 before (BC_2053-2055) and after (BCSD_2053-2055) spatial disaggregation under the RCP4.5 and RCP8.5 scenarios.

References

1. Gudmundsson L, Bremnes JB, Haugen JE, Engen-Skaugen T. Technical Note: Downscaling RCM precipitation to the station scale using statistical transformations–a comparison of methods. Hydrol Earth Syst Sci. 2012;16(9):3383-90.

2. Brekke L, Thrasher B, Maurer E, Pruitt T. Downscaled CMIP3 and CMIP5 climate projections: Release of downscaled CMIP5 climate projections, comparison with preceding information, and summary of user needs. US Department of the Interior, Bureau of Reclamation, Technical Services Center, Denver, Colorado, USA. 2013.
